# Therapeutic effect of titanium locking plate combined with suture anchor repair in proximal humeral fractures

**DOI:** 10.12669/pjms.41.1.11097

**Published:** 2025-01

**Authors:** Zhiwei Ye, Mengni Chen, Zhenfeng Huang

**Affiliations:** 1 Zhiwei Ye Department of Orthopedic Trauma, Wuhan Fourth Hospital, Wuhan, Hubei Province 430000, P.R. China; 2 Mengni Chen Department of Operating room, Tongji Hospital, Tongji Medical College of Hust, Wuhan, Hubei Province 430030, P.R. China; 3 Zhenfeng Huang Department of Orthopedic Trauma, Wuhan Fourth Hospital, Wuhan, Hubei Province 430000, P.R. China

**Keywords:** Titanium locking plate, Suture anchor, Proximal humeral fractures

## Abstract

**Objective:**

To explore the therapeutic effect of titanium locking plate combined with suture anchor (SA) repair in the treatment of proximal humeral fractures (PHF).

**Methods:**

This retrospective study was conducted by analyzing the clinical data of 113 patients with PHF admitted to Wuhan Fourth Hospital from March 2021 to October 2023. Among them, 55 patients underwent open reduction and internal fixation (OR/IF) using titanium locking plate (OR/IF group), and 58 patients underwent surgery with titanium locking plate combined with SA (SA group). Perioperative condition, treatment success rate, shoulder joint function before and after the surgery, shoulder joint range of motion, and incidence of complications were compared between the groups.

**Results:**

Surgery duration and the length of hospital stay of patients in the SA group were significantly shorter than those in the OR/IF group, and the intraoperative blood loss was significantly lower in the SA group than in the OR/IF group (P<0.05). In terms of treatment effect, the SA group was significantly higher than the OR/IF (P<0.05). After the surgery, muscle strength, pain levels, daily living activities, and shoulder joint range of motion scores of both groups improved, and the improvement was more significant in the SA group compared to the OR/IF group (P<0.05). After the surgery, the degrees of internal rotation, forward flexion, backward extension, and external rotation of the shoulder joints in both groups increased compared to pre- surgery levels, and were greater in the SA group compared to the OR/IF group (P<0.05). The incidence of complications in the SA group was lower than that in the OR/IF group (P<0.05).

**Conclusions:**

In patients with PHF, the combination of titanium locking plate and SA has a more significant therapeutic effect than that of titanium locking plate alone, which is associated with improved shoulder joint function and range of motion, and reduced incidence of complications.

## INTRODUCTION

Proximal humeral fractures (PHF) account for 5% of all fractures,^1^,^2^ and the rate continues to increase with the aging of the population, the increase of aerial work and traffic accidents.^1^-^3^ PHF have a significant impact on the daily activities of patients, significantly reducing their quality of life.^4^ Conservative treatment cycle for PHF is long and may result in significant functional loss and chronic pain, making it difficult to meet clinical needs.^5^ Therefore, surgery remains the preferred treatment for the disease.^5^,^6^

Surgical anatomical reduction and internal fixation can provide favorable conditions for early functional recovery of patients with PHF.^7^ While steel plate internal fixation is the most widely used method of surgical PHF repair, it is associated with certain risks such as screw cutting and easy breakage.^7^,^8^ Titanium locking plate have good biocompatibility and can provide a reliable fixation effect with their own interlocking structure, avoiding wear caused by friction between conventional steel plates and fractures.^9^ Suture anchors (SA) are also commonly used in fracture repair, which can form strong tension after implantation and accelerate fracture healing.^10^ Due to the high amount of cancellous bone in the proximal humerus, elderly patients often have varying degrees of osteoporosis, and the greater tuberosity fracture is mostly comminuted. SA fixations have been reported to have good outcomes for greater tuberosity fractures.^11^,^12^ Additionally, SA cause minimal damage to soft tissues and are resistant to pulling, which helps to ensure functional recovery.^10^,^13^

Although locking plate fixation and SA are widely used for PHF, there are few studies exploring the efficacy of titanium locking plate combined with SA for PHF. A recent report shows that the combination of locking plate and SA can restore function, minimize complications, and expedite recovery.^14^ Therefore, this study aimed to further confirm the efficacy of locking plate combined with SA for patients with PHF by retrospectively analyzing the clinical data of the patients who underwent surgical treatment in our hospital.

## METHODS

This retrospective study was conducted by analyzing the clinical data of patients with PHF admitted to Wuhan Fourth Hospital from March 2021 to October 2023. According to surgical records, patients who received OR/IF treatment with titanium locking plate were included in the OR/IF group, and patients receiving treatment with titanium locking plate combined with SA were included in the SA group.

### Ethical Approval:

The ethics committee of our hospital has reviewed and approved this retrospective study with number: KY2024-131-01, Date: June 24, 2024.

### Inclusion criteria:

- PHF, confirmed by CT, X-ray and other examinations.
- Age ≥ 18 years.
- Had indications for surgery, and underwent surgical treatment in our hospital.
- Complete clinical data.
- Closed fracture.

### Exclusion criteria:

- Patients with coagulation dysfunction.
- Patients with damage to important blood vessels and nerves.
- Patients with diseases that affect shoulder mobility prior to fracture.
- Patients with pathologic fractures or open fractures.
- Merged fractures in other parts of the body.
- Non-external violence-induced fractures.

### OR/IF group:

Titanium locking plate was used for OR/IF treatment. Patients were instructed to lie flat and maintain the forearm in a supinated position. Brachial plexus block anesthesia was initiated. Airbags and tourniquets were used to enter through the pectoralis major and deltoid muscles, strictly protecting the cephalic vein. If necessary, anterior deltoid muscle separation was done to effectively expose the proximal humerus and prevent axillary nerve damage. Manual reduction was performed according to the type and specific location of the fracture. Fracture was fixed with clover steel plate and T-shaped steel plate. Subsequently, internal fixation was implemented using steel plate screws.

### SA group:

Titanium locking plate combined with SA were used.^14^ Patients were instructed to lie flat and brachial plexus block anesthesia was initiated. Using pectoralis major and deltoid muscle approach, the affected shoulder was elevated using a sandbag, and an incision of about 12 cm was made on the outer side of the groove between the pectoralis major and deltoid muscles, separating the subcutaneous fat. Hemostasis of superficial veins was monitored. Subcutaneous fascia was separated, clarifying the course of the cephalic vein, bluntly separating the deltoid muscle fibers to protect the cephalic vein, and pulling the cephalic vein and muscle fibers together.

The anterior joint capsule was exposed, and cut open, exposing the fracture end, and removing the surrounding hematoma at the fracture end. Structures such as the biceps tendon and nodules were carefully located, and possible cases of cuff injury were identified. Soft tissue were cleaned, embedded at the fracture end, and the displacement of the fracture was identified. Flipped and collapsed humeral head was repositioned using periosteal stripping ions to ensure gentle movements. Care was taken to avoid extensive peeling of surrounding soft tissues. Using the intertubercular groove as a marker, large and small nodules of the humerus were reduced.

If it was difficult to distinguish the intertubercular groove in a comminuted fracture, the reduction of large and small nodules was carried out using the biceps brachii tendon as a reference. Each fracture block was temporarily fixed with Kirschner wires, and satisfactory reduction was confirmed by C-arm X-ray machine fluoroscopy. Two SA were inserted at the proximal end of the humerus, threaded through the tendon attachment site of the large and small nodules, tightened and knotted. Repair and suturing were implemented based on the condition of rotator cuff injury, and appropriate specifications for titanium locking plate was selected.

The suture was passed through the thread hole on the titanium plate, so that the highest position of the titanium plate was about 5mm away from the top of the large nodule and behind the groove between the nodules. Care was taken to avoid impact under the shoulder peak due to the high position of the titanium plate, and to prevent the blood supply to the long head tendon of the biceps brachii muscle and the ascending branch of the anterior humeral circumflex artery from being too close to the intertubercular sulcus. One screw (not tightened) was inserted into the proximal hole and the distal position of the titanium plate was adjusted appropriately.

One cancellous bone tension screw (not tightened) was inserted into the distal sliding hole, and the suture was tightened to avoid retraction of large and small nodules. Proximal humeral screw was then thoroughly inserted, distal tension screw and other locking screws were tightened. This way, the titanium plate fits more closely to the bone surface and corrects lateral displacement. Through C-arm X-ray machine fluoroscopy, it was determined that the screw tip was located approximately 0.5 cm below the articular cartilage. Special care was taken to ensure good grip and prevent the screw from cutting out of the joint surface, ensuring satisfactory position of the humeral distance screw on the titanium plate. After clarifying the stability and mobility of the shoulder joint, surgical incision was closed.

### Data collection:

- Baseline data: gender, age, affected side, cause of injury, Neer classification, fracture to surgical interval.
- Perioperative situation including surgical duration, intraoperative blood loss, and length of hospital stay.
- Treatment effect in the 8th week after the surgery was determined using X-ray to assess the reduction status that was classified as: Excellent-The anatomical alignment and alignment of the fracture ends are good, and there is no rotation of the humeral head; Good- Near and far fracture ends close to anatomical alignment, poor alignment effect, humeral head rotation; General-The rotation and inclination of the humeral head are all within < 15 °, with more than two-thirds of the distal and proximal fracture ends aligned and forming an angle of < 15 °; Poor-Failure to meet the above standards or even deterioration.
- Shoulder joint function at eight weeks post-surgery was assessed according to the Constant- Murley score.^15^,^16^ This evaluation includes muscle strength (25 scores), pain (15 scores), daily living activities (20 scores), and shoulder joint range of motion (40 scores). Higher score indicates better shoulder joint function.
- Shoulder joint range of motion in the 8th week after surgery, including degrees of internal rotation, forward flexion, backward extension, and external rotation.
- The incidence of complications including deformity healing, shoulder joint stiffness, subacromial impact, and limited abduction.

### Statistical Analysis:

Data were analyzed using SPSS version 25.0 (IBM Corp, Armonk, NY, USA). For continuous variables, mean and standard deviation (SD) were calculated. Paired *t*-tests were used to determine intra group preoperative and postoperative differences, while independent sample *t*-tests were used for intergroup comparisons before or after each surgery. For categorical variables, frequency distribution was provided and expressed as a percentage. The chi square test was used to compare categorical variables between two groups. A *p*-value less than 0.05 was considered to indicate a statistically significant difference.

## RESULTS

A total of 113 patients (including 65 males and 48 females) met the inclusion criteria for this study. Age of the patients ranged from 22 to 72 (mean ± SD, 44.36 ± 11.71) years. There were 55 cases in the OR/IF group and 58 cases in the SA group, with no significant difference in gender, age, affected side, cause of injury, Neer classification, and time from fracture to surgery between the two groups (*P*>0.05) (Table-I).

**Table-I:** Comparison of baseline data between two groups.

Baseline data	SA group (n=58)	OR/IF group (n=55)	t/χ^2^	P
Gender [n (%)]				
Male	32 (55.17)	33 (60.00)	0.269	0.604
Female	26 (44.83)	22 (40.00)		
Age (year)	43.86±10.35	44.89±13.06	-0.465	0.643
Affected side [n (%)]				
Left	31 (53.45)	32 (58.18)	0.256	0.613
Right	27 (46.55)	23 (41.82)		
Cause of injury [n (%)]			3.923	0.270
Falling from heights	6 (10.34)	9 (16.36)		
Traffic accidents	21 (36.21)	22 (40.00)		
Cycling injury	14 (24.14)	9 (16.36)		
Heavy object injury	17 (29.31)	15 (27.27)		
Neer classification [n (%)]				
II	29 (50.00)	34 (61.82)	1.598	0.206
III	29 (50.00)	21 (38.18)		
Time from fracture to surgery (day)	3.83±1.65	3.49±1.26	1.216	0.226

Surgery duration and the length of hospitalization of patients in the SA group were significantly shorter than those in the OR/IF group, and the intraoperative blood loss was significantly lower compared to the OR/IF group (*P*<0.05) (Table-II). The treatment effect of the SA group (93.10%, 54 of 58 cases) was significantly higher than that of the OR/IF group (80.00%, 44 of 55 cases) (*P*<0.05) (Table-III).

**Table-II:** Comparison of perioperative conditions between two groups.

Group	n	Surgery duration (minute)	Intraoperative blood loss (ml)	Length of hospital stay (day)
SA group	58	54.78±9.29	63.93±12.02	4.21±1.22
OR/IF group	55	62.58±8.18	74.02±11.87	5.49±1.36
*t*		-4.732	-4.486	-5.281
*P*		<0.001	<0.001	<0.001

**Table-III:** Comparison of treatment effects between two groups [n (%)].

Group	n	Excellent	Good	General	Poor	Excellent and good rate
SA group	58	45 (77.59)	9 (15.52)	4 (6.90)	0 (0.00)	54 (93.10)
OR/IF group	55	32 (58.18)	12 (21.82)	9 (16.36)	2 (3.64)	44 (80.00)
*χ* ^2^						4.210
*P*						0.040

Before the surgery, there was no significant difference in muscle strength, pain, daily living activities, and shoulder joint range of motion scores between the two groups (*P*>0.05). After the surgery, all these indexed significantly improved compared to before surgery, and were significantly higher in the SA group compared to the OR/IF group (*P*<0.05) (Table-IV).

**Table-IV:** Comparison of shoulder joint function before and after surgery between two groups (score).

Time	Group	n	Muscle strength	Pain	Daily living activities	Shoulder joint range of motion
Before surgery	SA group	58	17.40±2.18	6.26±1.38	11.26±1.86	18.40±2.74
	OR/IF group	55	16.89±2.32	5.95±1.37	11.98±2.16	17.64±2.97
	*t*		0.434	0.423	0.525	0.785
	*P*		0.665	0.673	0.601	0.434
After surgery	SA group	58	22.03±2.15^a^	11.21±1.70^a^	17.90±1.41^a^	32.09±3.46^a^
	OR/IF group	55	20.35±1.86^a^	9.62±1.87^a^	15.87±2.20^a^	28.51±4.32^a^
	*t*		10.797	10.860	6.431	6.315
	*P*		<0.001	<0.001	<0.001	<0.001

***Note:*** Compared with the same group before surgery,

a P<0.05.

There was no significant difference in the preoperative degree of internal rotation, forward flexion, backward extension, and external rotation between the two groups (*P*>0.05). After the surgery, the degree of internal rotation, forward flexion, backward extension, and external rotation of the shoulder joints in both groups significantly increased, and were markedly higher in the SA group compared to the OR/IF group (*P*<0.05) (Table-V). The incidence of complications in the SA group (5.17%) was significantly lower than that in the OR/IF group (18.18%) (*P*<0.05) (Table-VI).

**Table-V:** Comparison of shoulder joint mobility before and after surgery between two groups (°).

Time	Group	n	Internal rotation	Forward flexion	Backward extension	External rotation
Before surgery	SA group	58	17.88±3.02	30.86±3.36	32.62±7.26	24.69±5.10
	OR/IF group	55	18.45±3.67	31.36±4.25	31.40±8.04	25.47±5.99
	*t*		0.873	1.265	1.138	1.138
	*P*		0.384	0.208	0.258	0.258
After surgery	SA group	58	57.17±5.25^a^	130.00±15.45^a^	130.10±13.71^a^	81.40±10.70^a^
	OR/IF group	55	54.33±7.73^a^	122.00±11.67^a^	120.18±11.22^a^	76.87±10.14^a^
	*t*		6.498	7.607	5.071	5.071
	*P*		<0.001	<0.001	<0.001	<0.001

***Note:*** Compared with the same group before surgery,

a P<0.05.

**Table-VI:** Comparison of incidence rates of complications between two groups.

Group	n	Abnormal healing	Shoulder joint stiffness	subacromial impact	Limited abduction	Total incidence rate
SA group	58	0 (0.00)	1 (1.72)	0 (0.00)	2 (3.45)	3 (5.17)
OR/IF group	55	1 (1.82)	3 (5.45)	2 (3.64)	4 (7.27)	10 (18.18)
*χ* ^2^						4.693
*P*						0.030

## DISCUSSION

The results of this study show that the combination of titanium locking plate and SA is more conducive than surgery with titanium locking plate internal fixation in reducing surgical duration and trauma, shortening the time for functional rehabilitation, and achieving more significant therapeutic effects. Our results further confirmed that the combination of titanium locking plate and SA has high application value in the treatment of PHF, and is beneficial for restoring shoulder joint mobility and function, improving overall treatment efficacy, reducing complications, and ensuring treatment safety.^10^,^13^,^17^ Our results are consistent with the results by Li et al.^14^ It is believe that SA plays a significant role in the combination treatment. Kim YJ et al.^18^ demonstrated that compared to OR/IF with steel plates, patients treated with suture anchor fixation for PHF have shorter surgical time, better clinical outcomes, and fewer complications. Compared to traditional approach, the combined suture anchor technology can obtain greater fixation strength and fixation area, improve the anti-pull-out ability of the locking steel plate and screw, better resist traction forces generated by the deltoid muscle and rotator cuff on the large nodular fracture block during early postoperative shoulder joint activity training, and complement the advantages of the locking steel plate. By providing a strong internal fixation effect, this method allows to ensure that patients can undergo functional rehabilitation training early after the surgery.^18^,^19^

However, previous studies focused on locking steel plates, which is somewhat different from the titanium locking plate used in this study. The clinical value of titanium locking plate has been clinically proven.^20^,^21^ As shown in the study by Lian YS et al.,^20^ the use of titanium locking plate for the treatment of PHF can improve fracture healing and prevent complications, thus improving the quality of life. Shi ZK et al.^21^ also confirmed that the treatment of comminuted PHF in the elderly patients using titanium locking plates is associated with improved bone healing time, and better Neer score. These studies are consistent with our results. Conventional steel plate internal fixation surgery can cause significant damage to soft tissues. Moreover, during the treatment, it can seriously damage local blood flow status, increase the risk of humeral head necrosis, delayed fracture healing, and other complications.^7^,^8^ The initial stability of the titanium locking plate is strong, and it can provide good support for both the inner and outer walls, and can also produce a certain stress dispersion effect on the fracture end.^22^ In elderly patients with combined osteoporosis, the unique structure of the titanium locking plate can ensure that the entire implant has good resistance to extraction, avoiding further displacement and loss of reduction of the fracture. It provides good stability while minimizing soft tissue trauma, ensures that patients can undergo earlier functional training, timely restores joint function and range of motion, and reduces the risk of complications.^21^-^23^

In addition, SA used in our study can be fixed to the intact bone below the large tuberosity after the suture passes through the rotator cuff. The anchor nail has good biocompatibility and its material is absorbable. Therefore, it can be completely embedded in the cortex, which not only has a repairing and reducing effect on the shoulder sleeves, but also allows to cover, compress, and fix large nodules to prevent fracture displacement due to joint friction.^24^,^25^ Seppel G et al.^25^ indicated that in the treatment of shoulder joint fractures, the position of the threaded rivet should be maintained in the same plane as the rivet and the rivet thread to prevent the suture from being clamped into the fracture block and affecting the healing effect of the fracture. When fixing the suture, it is necessary to ensure appropriate elasticity, maintain a certain tension on the shoulder sleeve, and avoid excessive looseness that may affect fracture healing and shoulder joint function recovery.

Finally, this study confirmed that compared to simple OR/IF with titanium locking plate, titanium locking plate combined with SA have stronger resistance to torsion and bending, providing strong stability. Moreover, after insertion into the body, there is no need to bend the bone plate, which is conducive to reducing damage to the surrounding tissues of the fracture, reducing the risk of screw loosening, and ensuring fracture healing and functional rehabilitation.^26^,^27^ This can provide reference for the treatment of PHF.

### Limitations:

Firstly, this is a retrospective study comparing the results of two surgical fixation methods for treating PHF. In addition, in many cases, both OR/IF or titanium locking plate combined with wire rivets can be used. Another limitation is the small sample size that may lead to a certain selection bias. Finally, the impact of the two surgical methods on the long-term functional recovery of patients was not analyzed. In the future, large-scale randomized controlled studies with longer follow-up are needed to evaluate the value of titanium locking plate combined with SA in the treatment of PHF.

## CONCLUSION

In patients with PHF, the combination of titanium locking plate and SA has a more significant therapeutic effect than that of titanium locking plate alone, which is associated with improved shoulder joint function and range of motion, and reduced incidence of complications.

### Authors’ contributions:

**ZY** and **MC:** Study design, concept and manuscript writing.

**ZY**, **MC** and **ZH:** Were involved in data collection, data analysis, interpretation and critical review.

**ZH:** Was involved in the manuscript revision and validation.

All authors have read, approved the final manuscript and are responsible for the integrity of the study.
